# The Healthy Activity Program lay counsellor delivered treatment for severe depression in India: systematic development and randomised evaluation

**DOI:** 10.1192/bjp.bp.114.161075

**Published:** 2016-04

**Authors:** Neerja Chowdhary, Arpita Anand, Sona Dimidjian, Sachin Shinde, Benedict Weobong, Madhumitha Balaji, Steven D. Hollon, Atif Rahman, G. Terence Wilson, Helena Verdeli, Ricardo Araya, Michael King, Mark J. D. Jordans, Christopher Fairburn, Betty Kirkwood, Vikram Patel

**Affiliations:** **Neerja Chowdhary**, MD, Sangath, Goa, India and London School of Hygiene & Tropical Medicine, London, UK; **Arpita Anand**, MSc, MA, Sangath, Goa, India; **Sona Dimidjian**, PhD, University of Boulder, Boulder, Colorado, USA; **Sachin Shinde**, MPA, Sangath, Goa, India; **Benedict Weobong**, PhD, Sangath, Goa, India and London School of Hygiene & Tropical Medicine, London, UK; **Madhumitha Balaji**, MSc, Sangath, Goa, India; **Steve D. Hollon**, PhD, Vanderbilt University, Nashville, Tennessee, USA; **Atif Rahman**, PhD, University of Liverpool, Liverpool, UK; **G. Terence Wilson**, PhD, Rutgers University, New Brunswick, New Jersey, USA; **Helena Verdeli**, PhD, Teachers College, Columbia University, New York, USA; **Ricardo Araya**, PhD, London School of Hygiene & Tropical Medicine, London, UK; **Michael King**, PhD, University College, London, UK; **Mark J. D. Jordans**, PhD, King's College, London, UK; **Christopher Fairburn**, PhD, Oxford University, Oxford, UK; **Betty Kirkwood**, PhD, London School of Hygiene & Tropical Medicine, London, UK; **Vikram Patel**, FMedSi, Sangath, Goa, Centre for Chronic Conditions and Injuries, Public Health Foundation of India, New Delhi, India and London School of Hygiene & Tropical Medicine, London, UK

## Abstract

**Background**

Reducing the global treatment gap for mental disorders requires treatments that are economical, effective and culturally appropriate.

**Aims**

To describe a systematic approach to the development of a brief psychological treatment for patients with severe depression delivered by lay counsellors in primary healthcare.

**Method**

The treatment was developed in three stages using a variety of methods: (a) identifying potential strategies; (b) developing a theoretical framework; and (c) evaluating the acceptability, feasibility and effectiveness of the psychological treatment.

**Results**

The Healthy Activity Program (HAP) is delivered over 6–8 sessions and consists of behavioral activation as the core psychological framework with added emphasis on strategies such as problem-solving and activation of social networks. Key elements to improve acceptability and feasibility are also included. In an intention-to-treat analysis of a pilot randomised controlled trial (55 participants), the prevalence of depression (Beck Depression Inventory II ⩾19) after 2 months was lower in the HAP than the control arm (adjusted risk ratio = 0.55, 95% CI 0.32–0.94, *P* = 0.01).

**Conclusions**

Our systematic approach to the development of psychological treatments could be extended to other mental disorders. HAP is an acceptable and effective brief psychological treatment for severe depression delivered by lay counsellors in primary care.

The 2010 Global Burden of Disease reported that depression ranks second in the league table of years of life lived with disability^1^ with a mean population point prevalence of about 5%.^2^ Social factors, particularly those related to economic or social disadvantages such as low education and violence, are major determinants.^3^ Depression is associated with profound functional impairment, increased mortality and a range of other global health concerns (for example poor infant growth,^4^ diabetes, cardiovascular disease and HIV^5^). A key grand challenge in global mental health is to develop and evaluate psychological treatments that can be delivered economically and appropriately.^6^ There is a robust evidence base in high-income countries that supports the efficacy of antidepressant medication and structured psychological treatments, particularly for moderate and severe depression.^7^ However, most people with depression do not receive either of these treatments.^8,9^ This so-called treatment gap is close to 90% in some countries.^9^ Adapting ‘off the shelf’ treatments, which have been developed in ‘Western’ cultural contexts for delivery by mental health professionals, to low- and middle-income countries has been associated with low acceptability and delivery challenges.^10^ The scarcity of mental health specialists in low- and middle-income countries, and the fact that even fewer work in primary care settings where most patients with depression present, has led to a focus on the role of non-specialist health workers in delivering treatments.^11^ Task-sharing, in which provision of psychological treatments are moved from specialists to workers with fewer qualifications following task-specific training and close supervision, has been shown to be an effective healthcare strategy to address this supply-side barrier.^12^ Ensuring contextual appropriateness would be expected to enhance patient acceptability and address demand-side barriers.

The work described in this paper was carried out from October 2010 to September 2013 under the aegis of PREMIUM (a Program for Mental Health Interventions for Under-resourced Health systems) in India.^13^ The overall aim of the PREMIUM programme is to investigate a systematic, reproducible method for developing psychological treatments that incorporate global evidence, are contextually appropriate and can be delivered by non-specialist health workers. The specific objective of this paper is to describe the application of this systematic approach to the development of a brief psychological treatment for patients with severe depression delivered by lay counsellors in primary healthcare. By lay counsellor, we mean a person who has no professional qualification in mental healthcare although they may have other professional qualifications. Our work focused on developing a psychological treatment for three reasons. First, psychological treatments are comparable in effectiveness with antidepressants, have lower relapse rates and enhance recovery rates in antidepressant non-responders.^14–16^ Second, we observed low rates of adherence with antidepressants in our earlier research in India, even with adherence support measures provided by a case manager.^17^ Third, such treatments may be scalable in the context of the increased acceptability of counselling as a healthcare intervention in many countries, including India where this programme is being implemented. We focused on severe depression because evidence-based guidelines developed by the World Health Organization (WHO) and other regulatory bodies recommend structured psychological treatments for severe depression.^7,18^

## Method

**Fig. 1:**
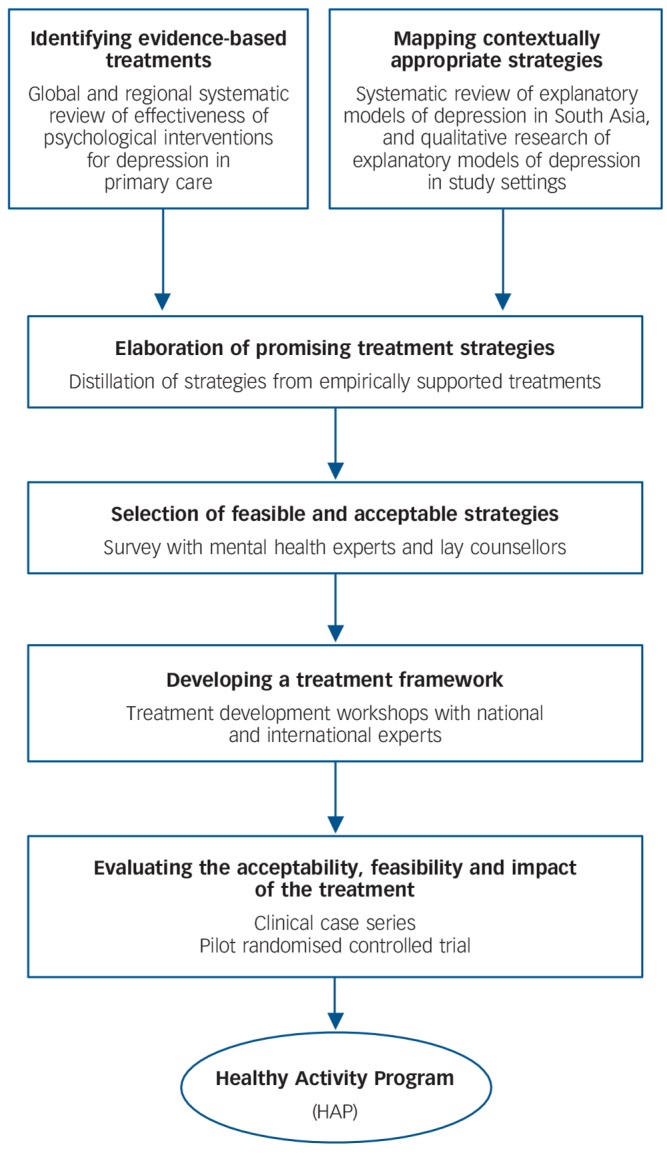
An overview of the psychological treatment development process.

The method we adopted arose out of our systematic review of methods used for cultural adaptations of psychological treatments for depression.^19^ We also were influenced by the approach of dismantling evidence-based psychological treatments to identify core treatment strategies that could be more easily task-shared with less qualified workers.^20^ The treatment development involved three stages (a) identifying potential treatment strategies; (b) developing a theoretical framework for the treatment; and (c) evaluating the acceptability, feasibility and impact of the treatment. The procedures in each stage were implemented as shown in Fig. 1. We present the methods and results of each stage below in order to demonstrate how the psychological treatment evolved through each of these stages of development. The field work was carried out in the states of Maharashtra and Goa, led by the non-governmental organisations Parivartan and Sangath, respectively. All research procedures with participants were approved by the institutional review boards of Sangath, the London School of Hygiene & Tropical Medicine and the Health Ministry Screening Committee of the Indian Council for Medical Research, and written informed consent was obtained from all participants.

### Stage 1: identifying potential treatment strategies

The goal of this stage was to identify promising strategies, drawn from both global and contextual evidence, for inclusion in the treatment. We kept in mind their likely acceptability to patients, effectiveness and ease of delivery by lay counsellors. This stage involved four steps: identification of evidence-based treatments (Step A); understanding the context for patients and families (Step B); distillation of promising treatment strategies from the global and contextual evidence (Step C); and finally selection of feasible and acceptable strategies (Step D).

#### Step A: identification of evidence-based treatments

Our review began with consideration of the WHO Mental Health Gap Action Programme (mhGAP) guidelines for evidence-based interventions for mental disorders in non-specialised healthcare settings.^7^ We supplemented these with searches of PubMed and PsycINFO (from 1 January 2009 to 1 January 2011). Concurrently, we carried out systematic reviews of regional (i.e. South Asian) literature on psychological treatments with searches of PubMed Central, IndMed, PsycINFO and PsycEXTRA (from 1 January 1990 to 1 January 2011). We also searched the ‘grey’ literature through hand searching reference lists of selected papers, contacting key informants and visits to key libraries in the region (search strategies and full list of key informants and libraries can be accessed in online Supplement DS1).

#### Step B: understanding the context for patients and families

In order to define the content and goals of the treatment, we explored the subjective experience of people with depression and their families in the study setting. This involved two methods: first, a systematic review of the literature on explanatory models of depression in South Asia; and second, in-depth interviews with patients with depression and family members. Findings from both methods were collated to address a number of questions: what are the explanatory models of people with depression and their family members; what are common self-help and coping strategies; which psychological and other treatments are sought and provided for depression, and what are the perceptions of their acceptability, feasibility and benefits; what is the acceptability and feasibility of delivery of such treatments by lay counsellors; and what are treatment expectations and desired outcomes? The detailed methods and findings of these studies have been published elsewhere.^21^

#### Step C: identification of treatment strategies from the global and contextual evidence

From the evidence-based treatments identified as described in Step A, we identified specific strategies following a procedure similar to the distillation process^20^ whereby interventions are conceptualised not as single units of analysis, but rather as composites of individual strategies, techniques or components that can be classified. We referred to the psychological treatment manuals and literature^22–26^ and consulted the psychological treatment experts in our investigators' group to ensure our list of strategies was accurate and complete. The following example illustrates this approach – the specific strategies identified from CBT were: psychoeducation, problem-solving, cognitive restructuring, activity scheduling and graded-task assignment. To this list of strategies, we added discrete contextually appropriate strategies identified in Step B.

#### Step D: selection of feasible and acceptable strategies

A consultation exercise was conducted in order to identify which of the strategies in our list would be most feasible for delivery by lay counsellors and acceptable by patients in the Indian context. Two groups of participants were invited: (a) mental health practitioners in India, chosen to represent a range of regions of the country and professional disciplines; and (b) potential lay counsellors, including hospital ward attendants, school counsellors and community multipurpose workers. Each respondent was asked to rate each strategy using a five-point Likert scale on four dimensions, with higher ratings indicating more positive perceptions: feasibility (it is possible for lay counsellors, with appropriate training and supervision, to deliver this strategy); acceptability (the strategy is regarded as suitable in the Indian cultural context and circumstances); effectiveness (the strategy brings about an important and positive change in the disorder); and risk of harm (there is a potential for harm or risk involved in a lay counsellor delivering this strategy). We combined mental health experts' and lay counsellors' ratings to produce an overall mean score for each strategy on the four dimensions.

### Stage 2: developing a treatment framework

The goal of this Stage 2 was to develop a conceptual framework for the treatment that was based on the component strategies identified in the previous stage. This stage involved six treatment development workshops with experienced mental health practitioners to assemble the strategies into a coherent treatment. The stage began with workshops with mental health experts in India (online Supplement DS2). Twenty-nine experts participated; they comprised psychologists (*n* = 11), psychiatrists (*n* = 13), social workers (*n* = 4) and a psychiatric nurse (*n* = 1). Ranking, pile sorting and scheduling methods were employed to finalise the list of strategies, to organise them into related groups, and structure them into a coherent treatment for delivery.^27^

### Stage 3: evaluating the acceptability, feasibility and impact of the treatment

The goal of this stage was to evaluate (a) the treatment's acceptability and feasibility in a case series and (b) its impact in a pilot RCT.

#### Evaluation of the acceptability and feasibility of the treatment

We carried out a case series over 9 months, in which both specialists and lay counsellors delivered the treatment (as described in the manual) to primary health centre attendees. Referrals from general practitioners and psychiatrists, and self-referred clients, were screened for inclusion using the Patient Health Questionnaire-9 (PHQ-9).^28^ This questionnaire had previously been validated in the study setting^29^ and patients with a score greater than 14, indicating moderately severe to severe depression, were offered the treatment delivered at weekly/fortnightly intervals. The specialists were four mental health professionals and one experienced therapist (non-specialist counsellor) who had been closely involved in the previous stages of the treatment development process and who were supervised by an international behavioural activation expert (S.D.). Nineteen lay counsellors were recruited from the local community and trained and supervised by the therapists, and were based in 11 primary health centres. Further details of the recruitment, training and competency of the lay counsellors are published elsewhere.^13,30^ Data were collected on the clinical process, including the engagement of patients, ease of use of specific strategies and modifications made, the experience of the phasing of the treatments, and other barriers experienced in the delivery of the treatment. There were seven serial focus group discussions with counsellors in four rounds; two each in the first three rounds and one in the fourth round. In each focus group discussion 8–10 counsellors participated. Three rounds of in-depth interviews with four supervisors were conducted (total 12 in-depth interviews/semi-structured interviews). Thirty patients were interviewed, 10 each for the following categories: dropped out, in-treatment and patients who have completed treatment.

**Analysis.** Data collection and analysis progressed iteratively, identifying and interpreting themes, leading to modifications to topic guides according to the emerging analysis. Grounded theory approach was used to analyse data. The guidelines used for the interviews and group discussions provided the *priori* framework for developing the themes and subthemes and data was coded in NVivo 8 qualitative data analysis software (QSR International, www.qsrinternational.com). We tallied simple frequencies for major themes, selected and analysed vivid and compelling examples of narrative extracts, and related these to the research questions.

#### Evaluating the impact of the HAP

The modified version of HAP was evaluated in a pilot RCT conducted over 5 months in eight primary health centres. Participants were primary health centre attendees recruited between August 2013 and October 2013 who scored over 14 on the PHQ-9 and who met eligibility criteria (aged above 17, resident in Goa and not requiring emergency treatment for any reason). The study was thus designed to include participants from varied social and educational backgrounds who attended primary care. As this was a pilot trial aimed primarily at assessing the acceptability and feasibility of the final treatment and to generate preliminary estimates of impact, no sample size estimations were carried out. Those who consented were randomly allocated in a 1:1 ratio to receive either enhanced usual care (EUC) or EUC plus HAP using a computer-generated allocation sequence, stratified by primary health centre and gender. The EUC consisted of the provision of screening results and mhGAP treatment guidelines to the primary health centre doctor. HAP was delivered by eight counsellors who satisfied competency criteria during assessments using structured role-plays.^30^ The primary outcomes were prevalence and severity of depression (based on the PHQ-9 and the Beck Depression Inventory (BDI-II)^31,32^ at 2 months post-enrolment; assessments were carried out by evaluators masked to group assignment. In addition, we assessed a range of process indicators (for example, number and duration of sessions required to deliver HAP). Intention-to-treat analyses adjusted for baseline PHQ-9 and that also accounted for clustering at the level of the primary health centre were carried out using Stata 11.

## Results

### Stage 1: potential treatment strategies identified

The global literature review undertaken in Step A to identify evidence-based treatments identified 33 papers (15 systematic reviews and 18 randomised control trials (RCTs)) and the regional review identified 23 papers (7 RCTs, 10 other study designs, 4 narrative reviews and 2 manuals/guidelines). The reference lists for the selected papers are provided in online Supplement DS3. The treatments identified through these two reviews were rated on two dimensions (i.e. the strength of the evidence of effectiveness and the generalisability of this evidence to the local context and feasibility of delivery by lay counsellors). Based on this approach, we selected the following treatments for further consideration: cognitive–behavioural therapy (CBT); interpersonal psychotherapy; problem-solving therapy; psychoeducation; behavioural activation; physical exercise; befriending; mindfulness-based cognitive therapy; acceptance commitment therapy, hypnosis, yoga and meditation.

Our findings from Step B, a systematic review of the literature on explanatory models of depression in South Asia and in-depth interviews with patients with depression and family members, were grouped under four broad categories: illness experiences, perceived impact, causal beliefs and self-help strategies. The experience of depression was characterised predominantly by physical aches and pains, stress (‘tension’), low mood, and negative and ruminative thoughts. Patients experienced disturbances in interpersonal relationships and occupational functioning, as well as stigma and discrimination. Family difficulties were perceived as the main cause of the problem, followed closely by financial difficulties or multiple social problems. To cope with distress, patients reported engaging in leisure activities and religious and spiritual practices, and seeking support from family and friends.

The final list of 22 treatment strategies we identified (Step C) are shown in Appendix 1. In the final step (Step D) of this stage of the process a total of 58 people, 15 experts and 43 lay counsellors, participated in the consultant exercise to select those strategies that were feasible and acceptable (online Table DS1). After ranking the strategies on the basis of scores and using *a priori* threshold (strategies with an overall score of 3.5 and above on the five-point Likert scale were retained), 15 of the 22 treatment strategies were taken forward to the next stage (online Table DS2).

### Stage 2: treatment framework formulated

Based on recommendations from the treatment development workshops, modifications were made to the list of 15 strategies notably: religious and spiritual practices were removed as these were considered to involve moral judgement and might induce guilt; support groups were not considered feasible to deliver in primary care settings; and reminiscence was perceived as likely to have limited effectiveness. The final scheduling led to a treatment consisting of four domains of strategies:

- engagement, which comprised psychoeducation, family psychoeducation and treatment planning – the aim of this domain was to educate and engage individuals and family members;
- activation, strategies for the delivery of tasks such as graded-task assignment, activity scheduling and physical exercise;
- need-based strategies, such as addressing interpersonal triggers, problem-solving, relaxation and enlisting social support tailored to the specific needs of individuals, and;
- social integration, as a way to reintegrate individuals into the community.

**Fig. 2:**
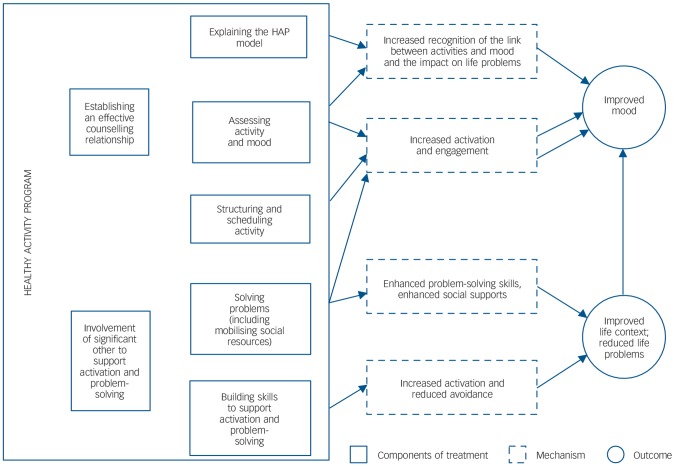
Modelling of intervention components and pathways to outcomes. HAP, Healthy Activity Program.

The international expert group recommended behavioural activation as the theoretical basis for the treatment because it provided the best fit, considering the culture and context in which the treatment was to be delivered and the fact that it captured most of the strategies in the list (Fig. 2). Following this decision, relevant treatment manuals were identified in consultation with the expert group. A content analysis of these manuals was done by assessing adequacy of the description of implementation of selected strategies, suitability for delivery by lay counsellors and the extent of adaptations needed for its use as the primary manual for the emerging treatment (online Supplement DS4). Based on this process, the *Behavioral Activation for Depression: A Clinician's Guide*^33^ was recommended.

This recommendation was reviewed in light of the feedback from patients and families in Stage 1 and determined to be an optimal fit for the setting. Specifically, the emphasis of behavioural activation on the importance of context in understanding the development and maintenance of depression was perceived to be responsive to primary patient concerns with family, financial and social problems, as well as stigma and discrimination. Additionally, the emphasis in behavioural activation on using activity and social context to promote recovery was closely aligned with patient reports of using activities and social support as coping strategies.

The *Behavioral Activation for Depression: A Clinician's Guide*^33^ manual was then adapted for further use in the following ways: simplification of the language; addition of culturally relevant case vignettes and scripts; and inclusion of other contextually appropriate strategies identified in the previous stage by incorporating procedures from other manuals, notably the one developed for the previous MANAS trial^34^ and from the *Engagement Session* manual (A. Zuckoff, personal communication, 2015) and Zuckoff *et al*,^35^ which provides a set of procedures to engage the patient in the treatment. The treatment resulting from this process was named the Healthy Activity Program (HAP).

### Stage 3: the acceptability, feasibility and impact of HAP

#### Acceptability and feasibility of HAP

Altogether, 271 patients participated in the case series (including 30 treated by the specialists). The modal patient was a middle-aged woman who had low educational attainment and was a home-maker. Nearly half (*n* = 133, 49%) dropped out, typically after the first session. There was a dose–response reduction in PHQ-9 scores measured in-session with the largest reduction being observed at session 6 (online Table DS3). The qualitative data were triangulated to address three key questions: (a) is HAP perceived to be useful by patients and counsellors, (b) what elements of HAP are perceived to be useful, and (c) what are the challenges faced in delivering HAP?

Briefly, regardless of their treatment status, most of the interviewed patients (*n* = 19/30) agreed that they found the treatment to be useful and that the counsellor helped them to address their problems. However, some patients who dropped out (*n* = 4/10) admitted that they had not understood what the treatment was about and were nervous about meeting the counsellor when asked to do so for the first time. Most patients who had engaged with the treatment (*n* = 16/20) found the following suggestions/advice very beneficial: completing the daily activity chart; spending time with friends and relatives; sharing one's thoughts and emotions; and doing things that the patient enjoyed.

Counsellor-related factors were described as promoting treatment engagement (*n* = 4), for example the counsellor was of a caring nature and provided continuing support. Patients who had dropped out usually reported doing so because of lack of benefit from the counselling and persistence of their health problems (*n* = 7/10). The commonest challenges to successfully completing treatment reported by patients (those who had engaged and those who had dropped out) were practical difficulties such as having to attend to work (*n* = 4) or difficulty in travelling to the primary health centre since there was no convenient public transport (*n* = 3). Some of the challenges reported by counsellors were difficulty in explaining the nature of treatment as many patients were not aware of the link between the stress in their daily lives and their health complaints; patients' reluctance to wait for the entire duration of the first session since they had not come to the primary health centre prepared for it; patients' failure to do the written homework between sessions; and, difficulty in engaging patients and maintaining session flow over the telephone.

Based on these findings, HAP was iteratively modified to enhance its contextual acceptability and feasibility for delivery by lay counsellors (online Supplement DS5). In particular, practical barriers in accessing primary care facilities led to the decision to choose home-based care as the main format of treatment delivery. Patients' social problems and somatic health concerns were given increased attention. Specifically, a list of resources and problem-solving strategies were created as scaffolding for the counsellors to utilise with patients. Resource materials for both patients and significant others were provided. Involvement of a significant other in the treatment sessions was encouraged. Use of written homework was replaced by in-session completion of activity monitoring charts. Identification of barriers to uptake of treatment, with discussion of possible solutions, was used in early sessions to enhance engagement. Appropriate language and metaphors were used to describe treatment components, for example using the term ‘tension’ instead of ‘depression’. The quality of the delivery was enhanced by provision of checklists for use by counsellors during sessions. Examples were step-by-step guidelines in dealing with difficult situations (especially high suicide risk) and off-the-shelf solutions derived from experiences in the clinical case series for dealing with social problems. Finally, simplification of therapeutic tools was emphasised, such as doing activity monitoring in blocks of time (morning, afternoon, night) rather than hourly and the use of icons to represent specific activities and emotions for patients with limited literacy to track activities.

#### Impact of the HAP

The modified version of HAP evaluated in the pilot RCT included 62 individuals who agreed to participate from a total of 142 eligible patients (participation rate 43.7%, online Table DS4). Common reasons for refusal to participate were lack of time (60%), need to gain permission from family before consenting (21%), not interested in the treatment (11%) and not residing in the area for the entire duration of the treatment (3%). The sample consisted mainly of middle-aged married women with low education. The outcome assessment was completed by 86% (24/28) and 91% (31/34) of participants in the HAP and EUC arms, respectively. The mean follow-up duration was 8.6 weeks (95% CI 8.2–9.1). There was no significant difference in baseline PHQ-9 score and sociodemographic profile between those who completed outcome evaluation and those who dropped out. Of all patients allocated to the HAP arm who were discharged (planned or unplanned (*n* = 19), 6 (33%) dropped out and 12 (67%) completed treatment; one patient was referred elsewhere. The remaining patients were still in treatment at the time of the 2-month follow-up. These process indicators showed improved treatment engagement compared with the earlier case series. Based on an intention-to-treat analysis (Table 1), the prevalence of depression (BDI-II ⩾19 at 2 months) was significantly lower in the HAP than EUC arm (adjusted risk ratio (RR) = 0.55, 95% CI 0.32–0.94, *P* = 0.01). In the HAP arm as compared with the EUC arm, treatment remission based on the PHQ-9 scores was higher (for PHQ <5 adjusted RR = 1.63, 95% CI 0.91–2.99, *P* = 0.09) and the severity of depressive symptoms was lower (mean difference on the BDI-II of 6.5, *P* = 0.1), although these differences did not reach statistical significance.

**Table 1:** Effect of the psychological treatment on depression outcomes at 2 months among adult primary health centre attendees in Goa, India

Outcome	Enhanced usual care arm (*n* = 31)	Healthy Activity Program arm (*n* = 24)	Adjusted risk ratio (95% CI)^a^	Adjusted risk difference or mean difference (95% CI)^a^	*P*
BDI ⩾19 at 2 months, *n* (%)	19 (61.3)	9 (37.5)	0.55 (0.32 to 0.94)	−28.7 (−50.8 to −6.6)	0.01
BDI score at 2 months, mean (s.d.)	22.8 (13.3)	16.5 (14.4)	–	−6.5 (−14.0 to 0.91)	0.10
Remission (PHQ <5) at 2 months, *n* (%)	9 (29.0)	11 (45.8)	1.63 (0.91 to 2.99)	20.3 (−3.2 to 43.8)	0.09

BDI, Beck Depression Inventory.

a. Results are adjusted for baseline Patient Health Questionnaire-9 (PHQ-9) score and primary health centre

## Discussion

### Key findings and interpretation

Our overall aim was to describe a systematic approach to the development of a contextually appropriate, scalable, brief psychological treatment for delivery by lay counsellors, illustrated by its application for the treatment of moderate/severe depression in primary healthcare in India. We identified potential evidence-based treatments from around the world; explored the subjective experiences of patients and their families; identified practical and acceptable delivery strategies in the local context; developed a theoretical framework; and subjected the resulting package to a proof of principle test in a large cohort. The final treatment package then was tested in a pilot RCT.

Our approach demonstrates four key points that may serve to guide future efforts.

First, it is important to take account of local strategies and health beliefs that are relevant to the context of treatments, for example the fact most patients do not regard their symptoms as related to ‘mental disorder’. Second, treatment strategies derived from global evidence have cross-cultural applicability, for example behavioural activation emerged as a highly relevant theoretical framework for the treatment. Third, the delivery of treatment must take into account local structural and cultural barriers that might reduce engagement, for example lack of familiarity with ‘talking’ treatments and both opportunity and direct costs in accessing such treatments. Fourth, developing an acceptable and feasible psychological treatment depends on following a systematic process that includes reviewing the global and contextual evidence, consulting with patients, families, mental health experts and lay counsellors, and evaluating the intervention in clinical case series and pilot studies.

A recent systematic review of cultural adaptations of psychological treatments for depression revealed that although some followed the steps advocated by the Medical Research Council framework for development of complex interventions,^19,36^ most did not report the method of adaptation. The methodology we have described in this paper, which incorporates evidence from treatments from around the world along with contextually appropriate practices and employs techniques such as distillation of strategies, offers a systematic method for the development of psychological treatments for delivery by non-specialist health workers. This approach guides treatment modification in an iterative manner and addresses acceptability and feasibility. In short, the PREMIUM psychological treatment development method addressed two elements simultaneously: (a) simplification so that lay counsellors could deliver it, and (b) cultural adaptation so that it was relevant to the target population, an approach consistent with that followed in another low-resource setting for children.^37^

### The HAP

Although the overarching aim of PREMIUM was to develop new treatments, which were built on global and contextual evidence, we observed that behavioural activation was a good match for our goals, indicating its cross-cultural acceptability and feasibility for delivery by non-specialist health workers. Our final treatment package is a brief structured psychological treatment for moderate/severe depressive symptoms that is delivered by lay counsellors in primary care. Its core strategies are treatment engagement, psychoeducation, behaviour activation, problem-solving, involvement of significant others, activation of social networks, relaxation, and techniques to improve interpersonal communication skills and address rumination.^38^ Figure 2 illustrates the various components of HAP and the proposed pathways through which the treatment outcomes are hypothesised to be achieved. A counselling relationship manual also assists lay counsellors to learn and enhance their core counselling skills.^39^ HAP is delivered in three phases (Appendix 2), with an optimum of six sessions (up to a maximum of eight), each session lasting 30–40 min, at weekly or fortnightly intervals. The patient's home or the primary health centre are the primary platforms for delivery and engagement of the significant other is encouraged as a means to promote treatment engagement.^39^

A number of challenges remain for the delivery of the treatment; these include: ensuring there are the resources needed for home-based delivery, especially for patients who come from distant/rural areas; maintaining counsellor competency over the long term; and retaining lay counsellors and preventing burnout. Our systematic approach to the development of psychological treatments needs to be extended to other mental disorders and replicated in other low-resource settings. The HAP requires further evaluation of its effectiveness and cost-effectiveness (currently in progress)^13^ and dissemination through m-health platforms.^40^

## Appendix 1

### Selection of psychological treatment strategies for the Healthy Activity Program (HAP)

**Table:**

Strategy	Effectiveness evidence^a^	Contextual appropriateness^b^	Perceived acceptability, feasibility, effectiveness and safety^c^
*Strategies included in HAP*			
Supportive counselling	+++	+++	++
Psychoeducation	+++	+++	++
Problem-solving	+++	+++	++
Enlisting social support	+++	+++	++
Relaxation	+++	+++	++
Physical exercise	+++	++	++
Addressing interpersonal triggers	+++	+++	++
Activity scheduling	+++	++	++
Graded task assignment	+++	++	++
Family psychoeducation		+++	++
Treatment planning	+++	+++	++

*Strategies not included in HAP*			
Value education		++	+
Reminiscence	+++		++
Cognitive restructuring	+++	+++	
Contextual functional analysis	+++	++	++
Focus on past experiences and relationships	+++		+
Radical acceptance	+++	+++	+
Addressing unconscious mechanisms	+++		
Family counselling	+		++
Support groups	+++	+++	++
Religious/spiritual practice		++	++

a. +++, Mental Health Gap Action Programme recommendation or at least one international systematic review or at least one randomised controlled trial (RCT) from South Asia; ++, at least one international RCT; +, any type of evidence from South Asia.

b. +++, Evidence of generalisability of international evidence or one RCT from region; ++, evidence from qualitative studies; +, other types of regional evidence.

c. ++, Composite score of both mental health professionals and lay counsellor ⩾3.5; +, composite score of both mental health professional or lay counsell or <3.5.

## Appendix 2

### The Healthy Activity Program (HAP) phases

**Table:**

Phase	Goals	Description
Early phase (delivered in 1–2 sessions)	Engaging and establishing an effective counselling relationship Helping patients understand HAP Eliciting commitment for counselling	Getting started Understanding the patient's problem using the HAP model and explaining this to the patient Getting commitment to the treatment Involving the significant other Addressing the patients' chief concerns (for example, distressing symptoms or pressing social problems) Addressing barriers to treatment engagement

Middle phase (delivered in 3–5 sessions)	Assessing activation targets and encouraging activation Identifying barriers to activation and learning how to overcome these Helping patients solve (or cope with) life problems	Assessment and activation strategies Problem-solving Other need-based strategies (for example, relaxation, addressing rumination, dealing with interpersonal triggers)

Ending phase (delivered in 1 session)	Reviewing and strengthening gains that the patient has made during treatment in order to prevent relapse.	Summarising key learning from the treatment Preparing for situations in the patient's life that may trigger depression in the future and generating plans to deal with such situations
